# Targeting BCL‐xL in Myeloid Malignancies: From Inhibitors to PROTAC

**DOI:** 10.1111/jcmm.71036

**Published:** 2026-04-06

**Authors:** Daniela Cilloni, Alessandro Ferrando, Francesco Frassoni

**Affiliations:** ^1^ Department of Clinical and Biological Sciences University of Turin Turin Italy

**Keywords:** BCL‐2, BCL‐2 inhibitors, BCL‐xL, PROTAC

## Abstract

Restoring apoptosis in malignant cells represents a central goal of anticancer therapy. Tumour cells often escape cell death by overexpressing anti‐apoptotic members of the BCL‐2 protein family, particularly BCL‐2, BCL‐xL, and MCL1. These proteins inhibit the intrinsic mitochondrial apoptotic pathway through intricate interactions with pro‐apoptotic partners and direct modulation of the mitochondrial outer membrane. Their pivotal role in cell survival has established them as attractive therapeutic targets. Over the past two decades, significant efforts have been devoted to developing selective small‐molecule inhibitors capable of neutralising these proteins and reactivating apoptosis. A first milestone was the discovery of ABT‐263 (navitoclax), a dual BCL‐2/BCL‐xL inhibitor. Building on this achievement, the development of venetoclax, a highly selective BCL‐2 inhibitor, marked a major breakthrough, demonstrating potent pro‐apoptotic activity and clinical efficacy in several leukaemia subtypes. Despite these advances, the design of inhibitors of BCL‐2 family members remains challenging, largely due to the structural characteristics of the BH3‐binding groove, which is both shallow and hydrophobic, complicating the identification of molecules with optimal binding affinity and selectivity. PROTACs targeting BCL‐xL may represent a promising future strategy, potentially overcoming the intrinsic limitations of small molecule inhibitors.

## 1 Introduction

Apoptosis is a tightly regulated process of programmed cell death that allows cells to maintain tissue homeostasis and prevent tumour development [1]. Two principal apoptotic pathways operate within the cell: the intrinsic and the extrinsic [2]. The extrinsic pathway is triggered by the activation of specific receptors on the cell membrane. These include Fas cell surface death receptor, TNF receptor 1 (TNFR1), and TRAIL death receptors. Subsequent activation of caspase 8 leads to activation of caspase 3, caspase 6 and caspase 7 [3]. The intrinsic mitochondrial pathway is mediated by various exogenous and endogenous stimuli that result in damage to the mitochondrial membrane. Several molecules play a key role in apoptosis induced through the intrinsic pathway, particularly proteins belonging to the B‐cell lymphoma‐2 (BCL‐2) family [4]. In addition to BCL‐2, the family of anti‐apoptotic proteins includes B‐cell lymphoma‐extra‐large (BCL‐xL) [5], Myeloid Cell Leukaemia sequence‐1 (MCL1) [6], BCL‐w [7], and BCL2A1 [8, 9]. All of these contain BH (BCL‐2 homology) domains, specifically BH1, BH2, BH3, and BH4 [10]. The anti‐apoptotic activity is primarily due to the first three, while BH4 binds proteins outside of the BCL‐2 family and regulates what is known as the non‐canonical activity of BCL‐2 and BCL‐xL [11].

Pro‐apoptotic proteins include BCL‐2–associated X protein (BAX), BCL‐2 antagonist killer (BAK), and BCL‐2–related ovarian killer (BOK), all of which contain the complete set of BH domains. In addition, the pro‐apoptotic subgroup comprises the so‐called BH3‐only proteins, named for retaining solely the BH3 domain, such as Bcl‐2 Interacting Mediator of cell death (BIM), BH3‐interacting domain death agonist (BID), BCL‐2 antagonist of cell death (BAD), NOXA, p53 upregulated modulator of apoptosis (PUMA), and BCL‐2‐modifying factor (BMF) [12].

Various stimuli, such as DNA damage caused by chemotherapy or radiation, elevated levels of reactive oxygen species (ROS), and the absence of growth factors, can trigger the intrinsic apoptotic pathway [13]. In response to death signals, BH3‐only proteins, sentinels of programmed cell death, are activated through various mechanisms, primarily including increased transcription, protein stabilisation, and post‐translational modifications [14, 15].

Once activated, specific BH3‐only proteins, such as BIM and BID, promote the activation of the pro‐apoptotic effectors BAX and BAK [16, 17, 18].

Proteins lacking a BH3 domain may also activate BAX and BAK. A notable example is p53, which, despite not containing a BH3 domain, has been shown to directly promote BAX and BAK activation [19, 20].

Upon activation, BAX translocates to the mitochondrial outer membrane, where BAK is already localised, and both undergo conformational changes. These structural rearrangements also mediate their interaction with anti‐apoptotic proteins such as BCL‐2 [18].

Following activation, BAX and BAK oligomerize to form pores in the outer mitochondrial membrane, resulting in mitochondrial outer membrane permeabilization (MOMP) and the release of cytochrome c, which serves as a ligand for apoptotic protease–activating factor 1 (APAF1). This event is accompanied by the release of additional pro‐apoptotic factors, such as Diablo (**D**irect **I**nhibitor of **A**poptosis‐binding **Lo**w pH), also known as Smac (**S**econd **M**itochondria‐derived **A**ctivator of **C**aspase) C and endonuclease G, which collectively contribute to the activation of the caspase cascade [18, 21].

**FIGURE 1:**
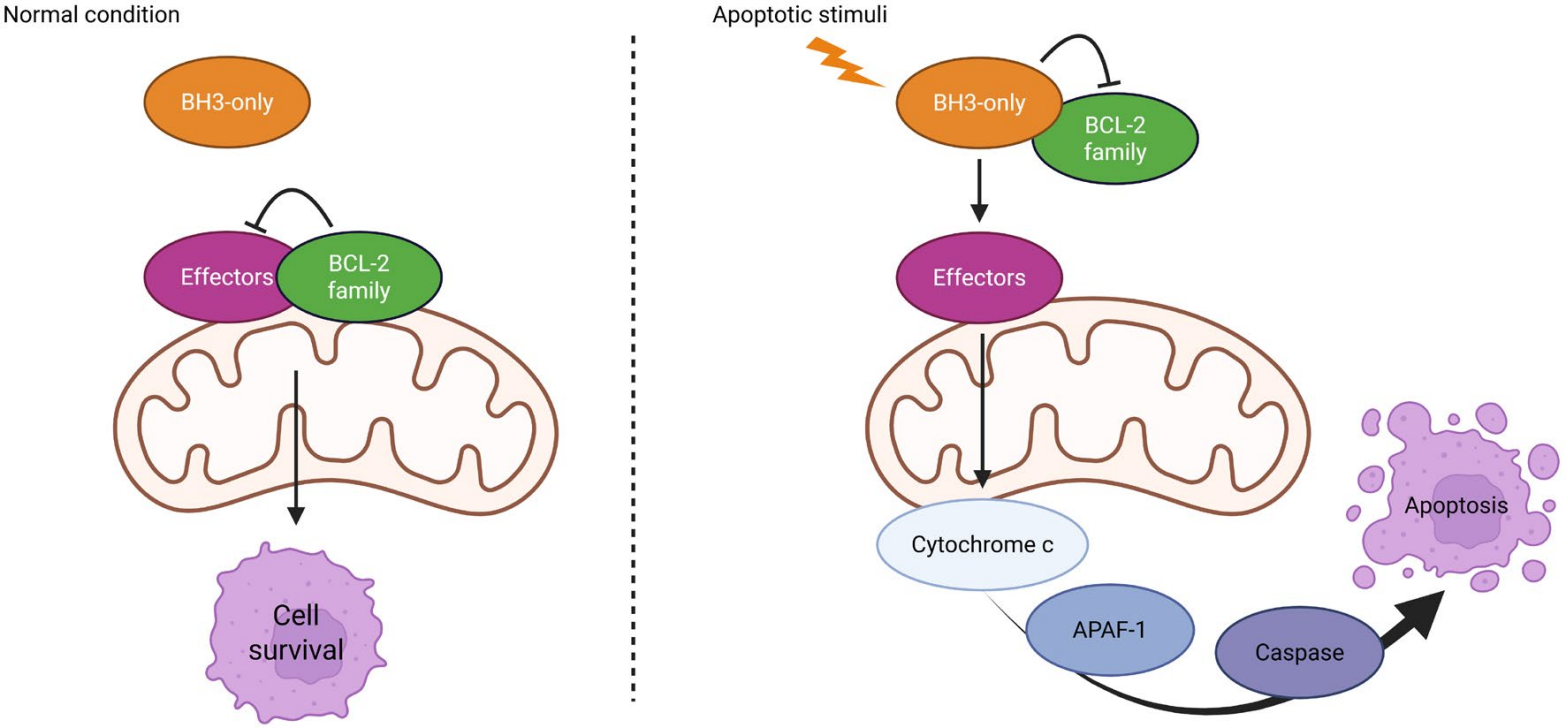
Regulation of mitochondrial apoptosis by BCL‐2 family proteins. Under normal conditions (left), anti‐apoptotic BCL‐2 family members prevent effector activation thus allowing cell survival. Upon apoptotic stimuli (right), BH3‐only proteins inhibit BCL‐2 family members and activate effectors, leading to mitochondrial outer membrane permeabilization, cytochrome c release, and subsequent activation of APAF1 and caspases, culminating in apoptosis.

Anti‐apoptotic proteins bind to the BH3 domains of BAX and BAK, thereby preventing their dimerization. The dynamic interplay between pro‐ and anti‐apoptotic BCL‐2 family members dictates the balance between survival and apoptosis, ultimately determining cell fate. MOMP constitutes the decisive event of the intrinsic pathway, irreversibly committing the cell to apoptosis. Accordingly, the regulation of MOMP by BCL‐2 family proteins represents a central checkpoint in the control of cell death. Cancer cells attempt to evade their fate and to escape drug‐induced apoptosis by upregulating one or more members of the BCL‐2 family [22], which has consequently drawn attention as a potential therapeutic target [23]. A graphical representation of the regulation of mitochondrial apoptosis by BCL‐2 family proteins is shown in Figure 1.

In this review, we aim to provide a concise overview of the biological role of BCL‐xL in the regulation of mitochondrial apoptosis and its contribution to the pathogenesis and progression of myeloid malignancies. We summarise current evidence on BCL‐xL dependency across different myeloid disease subtypes and discuss the available and emerging strategies to therapeutically target BCL‐xL, from small‐molecule BH3 mimetics to PROTAC‐based degraders and IAP‐recruiting approaches. Particular emphasis is placed on the potential clinical benefits and limitations of these agents, including on‐target platelet toxicity and mechanisms of resistance, with the goal of informing the future development and rational clinical use of BCL‐xL–directed therapies in myeloid neoplasms.

### 1.1 Expression of BCL‐2 Family Members in Haematological Malignancies

In 1984, the *BCL‐2* gene was first cloned from the breakpoint of the t(14;18) translocation in follicular lymphoma [4, 24, 25]. Since then, extensive studies have investigated the role of *BCL‐2* rearrangements and overexpression in haematological malignancies. BCL‐2 protein is overexpressed in approximately 90% of B‐cell lymphomas [26]. In acute myeloid leukaemia (AML), *BCL‐2* overexpression is detected in over 80% of patients at diagnosis and in nearly 95% of those with relapsed or refractory disease [27].

In this context, concomitant expression of BCL‐xL contributes to poor prognosis, largely through the promotion of chemoresistance [28]. Similarly, *BCL‐2* is overexpressed in 75%–80% of chronic lymphocytic leukaemia (CLL) cases [29]. In chronic myeloid leukaemia (CML), the overexpression of *BCL‐2* has been observed in leukaemic stem cells (LSC). In this setting, BCR::ABL can directly contribute to the upregulation of BCL‐xL and MCL‐1, providing additional mechanisms for cell survival [30]. Other malignancies rely predominantly on BCL‐xL or MCL‐1 to evade apoptosis and sustain cell survival [31]. In multiple myeloma (MM), for instance, *BCL‐2* expression is generally low, whereas high levels of *BCL2L1*, coding for BCL‐xL, and MCL‐1 are observed. Notably, MCL‐1 has been identified as a critical factor for MM cell survival and disease progression [32].

Recently, a relevant observation has been published by Kuusanmaki and colleagues who reported that acute myeloid leukaemia with erythroid or megakaryocytic differentiation depends on BCL‐xL rather than BCL‐2 for survival [33]. Notably, inhibition of BCL‐xL selectively induced apoptosis in blasts from patients with these AML subtypes and significantly reduced tumour burden in a mouse xenograft model [33].

Some years ago, we reported BCL‐xL overexpression in Philadelphia‐negative myeloproliferative neoplasms (MPNs), showing a progressive increase from essential thrombocythemia (ET) and polycythemia vera (PV) to myelofibrosis (MF). This pattern, independent of JAK2 mutational status, suggests a correlation between BCL‐xL upregulation and disease progression, highlighting its potential role as both a biomarker of aggressiveness and a therapeutic target [34].

Zhao and colleagues showed that BCR::ABL and mutant JAK2 block the BCL‐xL deamidation pathway, thereby suppressing the apoptotic response to DNA damage in primary cells from patients with CML or polycythemia vera [35].

*BCL2L1* overexpression has been documented not only in haematological malignancies but also across a wide range of solid tumours. In a study analysing more than 3000 samples from 26 different cancer types, *BCL2L1* and *MCL‐1* emerged as two of the most frequently upregulated genes, particularly in lung and breast cancers, where their expression correlates with poor prognosis and reduced survival [36].

### 1.2 BCL‐xL

BCL‐xL is one of the best‐characterised anti‐apoptotic proteins of the BCL‐2 family. Identified about a decade after BCL‐2, it was recognised as a key regulator of cell death [37].

BCL‐xL is the longer form of the protein produced by alternative splicing of the *Bcl‐x* gene, which is part of the BCL‐2 family [37]. BCL‐xL shares approximately 65% similarity with BCL‐2 and has a similar molecular weight of 26 KDa [37]. This protein is found in the outer membrane of mitochondria where it forms complexes with proteins like BAX, BAK, and BCL‐2, acting as an anti‐apoptotic regulator that prevents cytochrome C from being released into the cytosol.

The capacity of BCL‐xL to bind both BAX and BAK accounts for its anti‐apoptotic activity being approximately tenfold stronger than that of BCL‐2, which predominantly targets BAX [38]. When overexpressed at comparable levels, BCL‐xL is more effective than BCL‐2 in protecting cells from apoptosis induced by various stimuli. Tumours that rely on BCL‐xL, compared to those dependent on BCL‐2, have a higher metastatic capacity. Although both promote metastasis, BCL‐xL alone is sufficient to drive this process. It's likely that the anti‐apoptotic and invasive actions are supported by two different mechanisms [39].

Another intriguing aspect is that genetic alterations of BCL‐2 and BCL‐xL are rarely tumorigenic because their antiapoptotic function is usually compensated by an anti‐proliferative effect. BCL‐2 and BCL‐xL promote the resting G0 phase and delay G0‐S transition [40]. Dysregulated BCL‐xL and BCL‐2 activity can become oncogenic when associated with the amplification of other genes like MYC. The combination of these two abnormalities amplifies their oncogenic potential, resulting in a powerful tumorigenic combination [41].

The BCL‐x promoter region includes binding sites for several transcription factors, with signal transducers and activators of transcription (STATs), nuclear factor kappa B (NF‐κB), and members of the erythroblast transformation‐specific (ETS) family being particularly important. *BCL2L1* is a STAT5 target gene [42, 43]. Mutants such as JAK2^V617F, MPL^W515A/L/K, or BCR::ABL lead to constitutive JAK/STAT5 activation, resulting in upregulation of Bcl‐xL and protection of cells from apoptosis [44, 45, 46]. In leukaemias and solid tumours it was shown that activation of NF‐kB accounts for the upregulation of BCL‐xL [42, 45].

Recently, Afreen and colleagues [47] reported that *BCL2L1*–deficient human haematopoietic stem cells were markedly depleted following *BCL2L1* knockdown, whereas more mature granulocytic and monocytic cells showed less dependence on BCL‐xL expression. Notably, BCL‐xL deficiency could be fully compensated by BCL‐2 overexpression, while loss of its antagonist BIM failed to rescue either human erythroid cells or haematopoietic stem and progenitor cells. The authors further demonstrated that BCL‐xL expression is essential for the generation and maintenance of the human erythroid system, with “BCL‐xL addiction” arising already at the early stages of erythroid lineage commitment [47].

Silva and colleagues proposed that aberrant BCL‐xL expression may contribute to the erythropoietin‐independent survival of erythroid precursors in polycythemia vera [48].

*BCL2L1* is also constitutively expressed in the megakaryocytic lineage during megakaryopoiesis and is essential for platelet survival [49, 50]. Consistently, Kile and colleagues demonstrated that BCL‐xL is the key pro‐survival factor restraining apoptosis and ensuring platelet viability [51].

Debrincat and colleagues showed that, unlike BCL‐xL, BCL‐2 is dispensable for thrombopoiesis and platelet survival, at least in mice [52]. In 2002, Kirito and collegues [53] demonstrated that in normal megakaryocytes, *BCL2L1 expression* is regulated by thrombopoietin via JAK2 and PI3K signaling, leading to the activation of STAT5. In contrast, Guo and colleagues [54] demonstrated that JAK2^V617F^ mutation characteristic of myeloproliferative neoplasms enforces MCL‐1 transcription through STAT3 activation. Accordingly, treatment with JAK inhibitor I (JAKi‐I) attenuates STAT3 binding to the MCL‐1 promoter, thereby reducing both MCL‐1 mRNA and protein expression [54].

## 2 Compounds Targeting BCL‐xL

### 2.1 ABT‐737

As described above, the overexpression of the prosurvival BCL‐2 family members (BCL‐2, BCL‐xL, and MCL‐1) is commonly associated with tumour maintenance, progression, and chemoresistance. This knowledge has led to the identification of the BCL‐2 family members as excellent therapeutic targets in both solid tumours and various forms of leukaemia. The first compound developed with the intent to target these molecules was ABT‐737, a potent, small molecule BCL‐2 family protein inhibitor that occupies their BH3 binding groove [55]. ABT‐737 binds with high affinity to BCL‐xL, BCL‐2, and Bcl‐w. This compound demonstrated activity against small‐cell lung cancer (SCLC) and lymphoid malignancies. Although ABT‐737 potentiates the proapoptotic effects of several therapeutic agents used in the treatment of various cancers [56, 57, 58], its clinical development was hampered by several limitations. First, ABT‐737 has very poor oral bioavailability, making it unsuitable for patient use. Second, its inhibition leads to dose‐limiting thrombocytopenia, as platelets are highly dependent on BCL‐xL for survival. Finally, it does not target MCL‐1, a critical resistance factor in many hematologic and solid tumours, which further reduced its therapeutic potential.

### 2.2 ABT‐263 (Navitoclax)

In 2008 Tse and colleagues [59] reported the discovery of the first orally bioavailable BCL‐2/BCL‐xL inhibitor, navitoclax (ABT‐263), paving the way for a series of new drugs called BH3 mimetics.

Since myelofibrosis cells have been shown to rely on BCL‐xL for survival, a phase II trial was designed to evaluate the efficacy and safety of combining navitoclax with ruxolitinib in patients with myelofibrosis who had progressed on or had an inadequate response to ruxolitinib monotherapy (ClinicalTrials.gov identifier: NCT03222609) [60].

The phase III TRANSFORM‐1 trial was designed to evaluate navitoclax in combination with ruxolitinib versus ruxolitinib plus placebo in patients with untreated myelofibrosis (ClinicalTrials.gov identifier NCT04472598). An additional phase III trial named TRANSFORM‐2 was designed to evaluate the efficacy and safety of navitoclax plus ruxolitinib versus best available therapy in patients with relapsed or refractory myelofibrosis (ClinicalTrials.gov identifier NCT04468984). Preliminary data from the trials suggest that navitoclax is highly effective in reducing spleen volume. The main toxicity observed is thrombocytopenia, which is not associated with severe bleeding.

Navitoclax was evaluated in a phase I trial (ClinicalTrials.gov identifier NCT03181126) in patients with relapsed or refractory acute lymphoblastic leukaemia (ALL) in combination with chemotherapy [61]. To mitigate navitoclax‐induced thrombocytopenia, treatment was alternated with venetoclax. The overall complete remission rate was 60%, including responses in patients who had previously undergone haematopoietic cell transplantation or immunotherapy. The combination of venetoclax, low‐dose navitoclax, and chemotherapy was generally well tolerated and demonstrated encouraging efficacy in this heavily pretreated population.

No data are currently available on the use of navitoclax in acute myeloid leukaemia or myelodysplastic neoplasms (MDS). A phase Ib trial is ongoing to find the side effect and best dose of navitoclax when given together with venetoclax and decitabine in treating patients with acute myeloid leukaemia relapsed or refractory after previous treatment with venetoclax (ClinicalTrials.gov identifier: NCT05222984). An additional A phase Ib/II trial is ongoing to evaluate escalating doses of Navitoclax in combination with standard dose venetoclax and hypomethylating agents (HMA) in patients with relapsed or refractory high risk MDS HR‐MDS after failure of HMA (ClinicalTrials.gov identifier: NCT05564650). The most extensive clinical experience with navitoclax has been in chronic lymphocytic leukaemia (CLL). Several clinical trials are ongoing, and data from published studies are already available. In particular, Kipps and colleagues reported that in previously untreated patients, navitoclax combined with rituximab was superior to rituximab alone and resulted in prolonged progression‐free survival, with treatment extending beyond 12 weeks [62] (ClinicalTrials.gov identifier: NCT01087151).

## 3 Additional Drugs Targeting BCL‐xL

The development of selective BCL‐xL inhibitors or dual BCL‐2/BCL‐xL inhibitors has been hampered by dose‐limiting platelet toxicity, which is unlikely to be overcome by simply optimising BH3‐mimetic small‐molecule inhibitors. Several strategies are being investigated to expand the therapeutic window, and these approaches are currently under clinical evaluation.

**APG‐1252**, developed by Ascentage Pharma, is a BH3‐mimetic, phosphate prodrug derived from a dual BCL‐2/BCL‐XL inhibitor, which has been developed to reduce platelets exposure [63]. APG‐1252 displayed lower permeability in platelets compared with cancer cells. Once internalised into target cells, it is converted into its active metabolite APG‐1252‐M1. Both APG‐1252 and APG‐1252‐M1 exhibited high affinity for BCL‐2 and BCL‐xL; however, APG‐1252‐M1 demonstrated approximately 10‐fold higher cytotoxic activity than APG‐1252 in lung cancer tumour cells [64].

**AZD‐0466** is a drug that utilises a dendrimer‐based delivery system, in the attempt to reduce toxicity. AZD‐0466 is a BH3‐mimetic, dual BCL‐xL/BCL‐2 inhibitor derived for AZD4320 by conjugation to PEGylated poly‐lysine units to optimise its release rate and enhance delivery to target cells. This drug has been tested in advanced haematological malignancies (ClinicalTrials.gov identifier NCT04865419).

A different strategy has been used for developing ABBV‐155, a BCL‐xL targeting antibody‐drug conjugate (ADC) which efficiently delivers the active drug in tumour cells expressing B7H3. This approach is expected to minimise drug exposure to normal tissues, thereby reducing the potential for off‐target toxicity. This drug is in clinical trial for treating advanced solid tumours (ClinicalTrials.gov identifier NCT03595059).

## 4 BCL‐2 Inhibitors

In parallel with the development of BCL‐XL inhibitors, the clinical development of BCL‐2–selective inhibitors has provided a crucial proof‐of‐concept for pharmacologic targeting of the mitochondrial apoptotic machinery. Venetoclax (ABT‐199), the first‐in‐class, highly selective BCL‐2 inhibitor, shows a remarkably strong preference for BCL‐2, binding to this protein in vitro with an affinity over a thousand‐fold higher than its affinity for BCL‐XL or BCL‐w [65].

Venetoclax has shown impressive single‐agent and combination activity in B‐cell malignancies [66] and, more recently, has reshaped the treatment landscape of older or unfit patients with AML when combined with hypomethylating agents [67]. In this setting, venetoclax exploits the BCL‐2 dependency of leukaemic stem and progenitor cells, but its efficacy is limited in disease subsets that preferentially rely on alternative anti‐apoptotic proteins, particularly BCL‐xL or MCL‐1 [33].

Next‐generation BCL‐2 inhibitors such as sonrotoclax (BGB‐11417) [68] have been designed to further increase potency and selectivity for BCL‐2, with the aim of deepening responses and overcoming some mechanisms of venetoclax resistance while maintaining a more favourable safety profile compared with dual BCL‐2/BCL‐xL inhibitors like navitoclax, in which on‐target thrombocytopenia is dose‐limiting. BGB‐11417 exhibits strong in vitro and in vivo inhibitory activity against both wild type WT BCL‐2 and G101V mutant.

The contrasting patterns of BCL‐2 versus BCL‐xL dependency observed across hematologic malignancies underscore the need for a rational, lineage and genotype informed use of BH3 mimetics, and provide the biological rationale for selectively targeting BCL‐xL through improved inhibitors or degraders as a complementary strategy to BCL‐2 inhibition, particularly in myeloid neoplasms characterised by venetoclax resistance and dominant BCL‐xL addiction.

## 5 PROTAC Technology

Many proteins that play a critical role in tumour development are considered undruggable because they lack a suitable binding pocket for conventional small molecule inhibitors. The utility of these molecules is further limited by their reliance on occupancy‐based pharmacology, which requires prolonged exposure and high local concentrations; conditions that can increase toxicity and promote the emergence of drug resistance.

**FIGURE 2:**
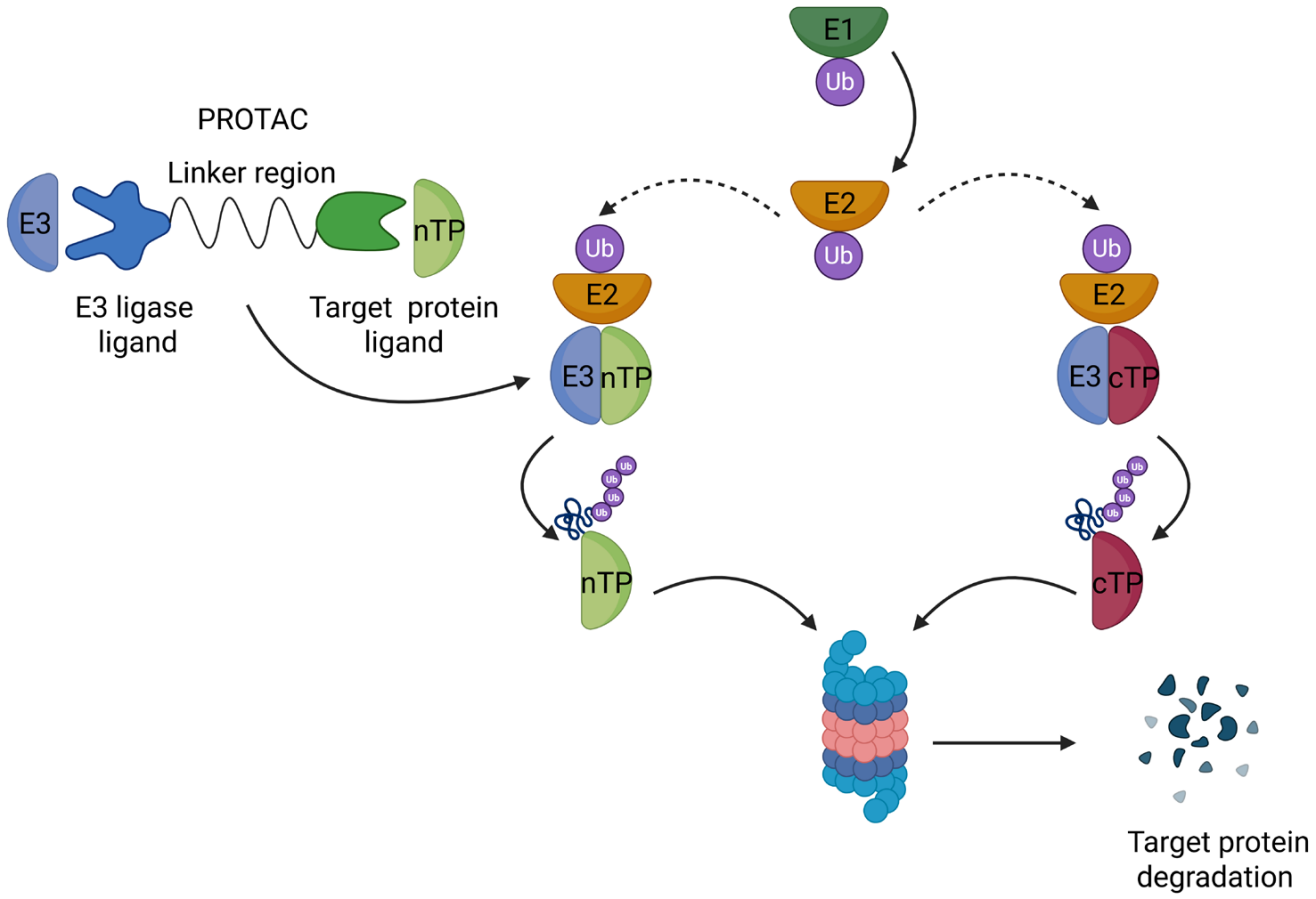
PROTAC‐mediated protein degradation via the ubiquitin proteasome system. PROTAC molecules consist of two ligands connected by a linker: one binds an E3 ubiquitin ligase, the other the target protein (TP). Ubiquitin‐activating enzymes (E1) catalyse the adenylation of ubiquitin, after which the activated ubiquitin is transferred to a ubiquitin‐conjugating enzyme (E2). Ubiquitin ligases (E3) recognise the TP, bridging it with the E2–ubiquitin complex and facilitating the transfer of ubiquitin to the substrate. Subsequent ubiquitin molecules can be attached, generating polyubiquitin chains whose specific linkage patterns dictate distinct downstream consequences for the protein's destiny. Once tagged, the ubiquitinated protein is recognised by specialised proteasomal subunits through ubiquitin‐binding receptors or associated domains. This recognition triggers protein unfolding and translocation into the proteasome's catalytic core, where degradation occurs. Finally, deubiquitinating enzymes recycle ubiquitin molecules, allowing their reuse in further ubiquitination cycles. cTP, Canonical target protein; nTP, non‐canonical target protein.

Proteolysis targeting chimeras (PROTACs) are innovative molecules developed to target specific proteins of interest, mainly oncoproteins [69]. As shown in Figure 2, PROTACs induce selective ubiquitination and subsequent proteasomal degradation of target proteins leading to a significant reduction of their expression levels. They can be considered a revolutionary advance due to their ability to eliminate previously undruggable proteins including transcription factors and scaffolding proteins.

PROTACs exploit the physiological protein degradation system. Within cells, proteins are tagged for degradation through the covalent attachment of ubiquitin chains, which serve as signals for the 26S proteasome. This process involves a cascade of three enzyme classes (E1–E3). Ubiquitin‐activating enzymes (E1) adenylate ubiquitin and transfer it to conjugating enzymes (E2), while ubiquitin ligases (E3) recognise the target protein and facilitate the transfer of ubiquitin from the E2–ubiquitin complex onto the substrate. Polyubiquitinated proteins are then recognised by dedicated proteasomal subunits, unfolded, and translocated into the catalytic core for degradation. Deubiquitinating enzymes subsequently release ubiquitin molecules for reuse. The remarkable diversity of E3 ligases (more than 600 E3 ligases have been identified in humans) confers substrate specificity and provides extensive opportunities for therapeutic modulation [70, 71].

PROTACs are heterobifunctional molecules composed of three essential elements: (i) a target‐binding ligand (“bait”) that recognises and binds the protein of interest, (ii) an E3 ligase–recruiting ligand, and (iii) a chemical linker that connects the two. By pairing a bait with high affinity and specificity for the target protein with an E3 ligase that is preferentially expressed in the desired cell type, PROTACs can achieve remarkable selectivity. This strategic combination enables cell‐type–restricted degradation of the target, minimises off‐target effects, and reduces adverse events relative to traditional small‐molecule inhibitors. The PROTAC mediated E3 ligase recruitment leads to polyubiquitination of the target protein and its subsequent degradation through the ubiquitin‐proteasome system. PROTAC, after dissociation from the target protein, is recycled and ready to bind another copy of the target protein [72, 73, 74].

The first generation PROTACs were peptide based with the limits of poor chemical stability and cell permeability that limited the clinical development [75]. Second generation PROTACs are based on small molecules E3 ligase ligands. Many oncoproteins have been effectively targeted with this technology including anaplastic lymphoma kinase (ALK), cyclin dependent kinase (CDK) and bromodomain and extraterminal domain protein (BET) [76, 77, 78, 79].

The key technological advantage of PROTACs lies in their ability to selectively degrade target proteins with high efficiency. By removing rather than merely inhibiting proteins, PROTACs have the potential to reduce the off‐target effects commonly associated with traditional drugs. Moreover, they exhibit favorable pharmacokinetic properties, including absorption, distribution, metabolism, and excretion [79]. Based on this exceptional therapeutic potential, many E3 ubiquitin ligases have been examined and evaluated. However, only a few of these have actually been used to build PROTACs [80]. von Hippel–Lindau (VHL) and Cereblon (CRBN) remain the most commonly used E3 ubiquitin ligases to date. The advantage of these proteins is mainly that they are expressed across multiple cell types, facilitating protein targeting in different tissues. Nevertheless, the next generation of PROTACs will likely leverage the diversity of E3 ligases to achieve greater target‐specific functionality. Employing E3 ligases restricted to a limited set of cell subtypes offers the opportunity to improve tissue selectivity while reducing off‐target toxicity. In addition, E3 ligases that are uniquely present or overexpressed in malignant cells provide an attractive strategy for targeting essential wild‐type proteins in a highly selective cellular context.

Over 5000 PROTACs, targeting more than 300 different proteins, have been designed [81].

An example is provided by PROTAC based on crystal structures of JAK2 kinase domain in complex with ruxolitinib and baricitinib. This PROTACs targeting JAKs showed in vivo activity in *CRLF2*r acute lymphoblastic leukaemia (ALL) [82].

Many protein degraders have already moved to the clinic and are in phase I–III clinical trials for solid cancers and for different types of haematological malignancies [74].

PROTAC technology is being explored to selectively degrade BCL‐xL in cancer cells, leading to the development of the clinical‐stage drug candidate DT2216.

## 6 PROTAC Degrader of BCL‐xL

The first PROTAC specifically designed to target BCL‐xL was reported in 2019, marking a significant advance in the field of targeted protein degradation [83]. This molecule, DT2216, was rationally engineered to exploit the Von Hippel–Lindau (VHL) E3 ligase which is minimally expressed in platelets, to achieve selective degradation of BCL‐xL using navitoclax‐piperazine (HY‐44432) derived ligand as the targeting warhead. By recruiting VHL, DT2216 effectively spared platelets and overcame the dose‐limiting thrombocytopenia associated with conventional BCL‐xL inhibitors. DT2216 demonstrated potent antitumor activity in preclinical models with an improved therapeutic window, establishing a strong proof‐of‐concept for PROTAC‐mediated targeting of anti‐apoptotic BCL‐2 family members. Building on these findings, Wang and colleagues [84] recently evaluated DT2216 in post‐MPN acute myeloid leukaemia harbouring JAK2 mutations, confirming that VHL‐mediated degradation of BCL‐xL can provide a therapeutic advantage while minimising platelet toxicity, owing to the limited expression of VHL in platelets. The authors demonstrated that *BCL‐xL* is significantly overexpressed in post‐MPN AML compared with de novo AML, reflecting a pronounced dependence of leukaemic cells on this anti‐apoptotic factor. Notably, this survival dependency was especially prominent in AML cells with concurrent *TP53* mutations, which are otherwise associated with therapeutic resistance and poor prognosis. The authors further demonstrated that DT2216 reduces the viability of haematopoietic stem and progenitor cells and effectively suppresses the growth of *JAK2‐*mutated AML cells. The findings suggest that DT2216 may represent a promising and targeted therapeutic strategy for this high‐risk AML subset [84].

Following the development of DT2216, Zhang and colleagues reported XZ739, an ABT‐263‐derived BCL‐xL degrader that, unlike DT2216, recruits the Cereblon E3 ligase. The choice of the E3 ligase is based on the fact that both CRBN and VHL are expressed at negligible levels in platelets [85].

In the same year, Pal and colleagues reported the development of a novel BCL‐xL PROTAC, designated PZ703b. The distinctive feature of this compound is its hybrid mechanism of action: while PZ703b efficiently induces BCL‐xL degradation thanks to ABT‐263 targeting, it inhibits but does not degrade BCL‐2, representing an unprecedented dual‐targeting strategy among PROTAC molecules. Consequently, PZ703b displays remarkable potency against BCL‐xL‐dependent, BCL‐2‐dependent, and dual‐dependent tumour cells in a strictly VHL‐dependent manner [86].

More recently, Zheng and colleagues designed, synthesized, and evaluated a novel series of BCL‐xL PROTACs by linking the selective inhibitor A‐1331852 to either VHL or CRBN ligands. These degraders showed enhanced cytotoxicity against BCL‐xL–dependent MOLT‐4 T‐ALL cells relative to DT2216, while sparing BCL‐2. Notably, compound XZ338 emerged as the most potent BCL‐xL degrader to date, exploiting A‐1331852 mediated BCL‐xL targeting; it reached 20‐fold greater activity than DT2216 in MOLT‐4 cells and about 90‐fold selectivity over human platelets [87].

Very recently, Poddar and colleagues [88] reported the development of a new generation of ABT‐263‐based PROTACs targeting BCL‐xL/BCL‐w that use the bis(sulfonyl)benzene ring of ABT‐263 as a linker vector. These degraders induced efficient degradation of BCL‐xL/BCL‐w while mitigating platelet toxicity. This work illustrates a rational approach to transform navitoclax derivatives into safer and more effective therapeutics for targeting anti‐apoptotic BCL‐2 family proteins.

## 7 Challenges for PROTAC Efficacy: Resistance Mechanisms and Toxicity Profiles

Although PROTACs offer unique advantages over occupancy‐driven inhibitors, several mechanisms of acquired and intrinsic resistance have been documented and are directly relevant for the development of BCL‐2 family degraders in leukaemia. A prominent resistance mechanism involves alterations in the recruited E3 ubiquitin ligase [89]. Loss of expression, downregulation, or mutation of key PROTAC‐engaged ligases (e.g., VHL or CRBN) can abolish ternary complex formation and prevent ubiquitination of the target protein. Selective pressure from chronic PROTAC exposure can also drive transcriptional remodelling of the ubiquitin–proteasome system, enabling cancer cells to escape degradation. Additionally, impairment of downstream degradative machinery, such as proteasome dysfunction or altered deubiquitinase activity, has been shown to attenuate PROTAC efficacy [90, 91].

Resistance may also arise through compensatory regulation of apoptosis pathways. Degradation of BCL‐xL or BCL‐2 can trigger adaptive upregulation of alternative anti‐apoptotic proteins, particularly MCL‐1, which frequently mediates therapeutic escape in hematologic malignancies. Similarly, activation of pro‐survival signalling pathways (e.g., NF‐κB, PI3K/AKT) can blunt the apoptotic response to BCL‐xL–directed degraders. These compensatory mechanisms highlight the importance of understanding the dynamic rewiring of mitochondrial apoptosis and may justify combination strategies involving MCL‐1 inhibitors, BH3 mimetics, or agents that neutralise adaptive signalling.

Toxicities remain a critical consideration in the translational development of PROTACs. Because PROTAC activity depends on the endogenous distribution of E3 ligases, tissue‐specific expression patterns (https://www.proteinatlas.org/ENSG00000113851) can result in on‐target toxicity in sensitive cell populations. For example, VHL‐ and CRBN‐recruiting degraders can inadvertently target proteins in non‐malignant tissues where these ligases are abundantly expressed [92]. Hepatotoxicity has been observed in preclinical models, potentially due to high hepatic E3 ligase levels or the accumulation of lipophilic PROTAC molecules with prolonged hepatic retention [93]. Immune‐related effects have also been reported, including cytokine modulation and alterations in antigen presentation, reflecting the complex immunologic roles of proteasome and E3 ligase pathways. Furthermore, some degraders may destabilise E3 ligases themselves or trigger auto‐ubiquitination, creating undesired impacts on cellular homeostasis.

Overall, while PROTACs hold significant therapeutic promise, a comprehensive understanding of E3 ligase biology, adaptive survival signalling, and tissue‐specific toxicities is essential for their safe and effective clinical translation. Continued mechanistic studies, profiling of resistance signatures, and careful biomarker‐driven patient selection will be key to fully harnessing the potential of targeted protein degradation in leukaemia and beyond.

## 8 Beyond PROTACs: SNIPERs

Two main limitations can hamper PROTAC‐based approaches: the emergence of resistance‐conferring mutations in recruited E3 ligases and the downregulation or low expression of these ligases in target cells. To address these barriers, Zhang and colleagues [94] developed inhibitor of apoptosis protein (IAP)–based degraders of BCL‐xL. In humans, eight IAP family members are expressed, each characterised by baculovirus IAP repeat (BIR) domains, zinc‐binding regions, a ubiquitin‐associated domain, and a RING finger domain that confers E3 ligase activity. Specific and Non‐genetic IAP‐dependent Protein Erasers (SNIPERs) represent a distinct class of targeted protein degraders that harness IAPs as the recruited E3 ligases. Structurally analogous to PROTACs, SNIPERs are heterobifunctional molecules composed of a ligand that binds the protein of interest and an IAP‐binding moiety, typically derived from bestatin, LCL161, or related BIR‐binding derivatives, connected via a chemical linker. These IAP‐recruiting ligands bind to a conserved region within the BIR domain shared across multiple IAPs. As a result, cancer cells would need to simultaneously disrupt several IAP family members to fully evade degradation, an evolutionarily costly strategy because IAP loss compromises essential survival pathways. By engaging both the target protein and the IAP E3 ligase (such as cIAP1), SNIPERs induce proximity‐driven ubiquitination and proteasomal degradation.

Importantly, whereas VHL or CRBN can be downregulated or mutated under therapeutic pressure, IAPs often exhibit the opposite behaviour. Their expression is frequently upregulated by oncogenic signalling, stabilised through NF‐κB activation, and tightly linked to pro‐survival and pro‐proliferative pathways. Because IAPs actively contribute to tumour fitness, malignant cells tend to preserve or even enhance their expression, making them a reliable and robust degradation scaffold for SNIPER‐based strategies.

SNIPERs have been successfully applied to degrade a broad range of proteins, including BCR::ABL, bromodomain‐containing proteins, nuclear factors, and other oncogenic targets [95, 96]. Although challenges such as tissue‐specific variability in IAP abundance and the intrinsic instability of some IAP binders remain to be addressed, ongoing optimization of ligand design and linker architecture continues to enhance their efficiency and specificity. Collectively, IAP‐based degraders broaden the degrader toolbox and offer a promising strategy to bypass key limitations associated with classical PROTAC technologies.

**Table:** 

Molecule	Target	Mechanism	E3 (PROTAC)	Trial
Navitoclax (ABT‐263)	BCL‐xL/BCL‐2	BH3 mimetic	—	NCT03222609 NCT04472598 NCT04468984 NCT03181126 NCT05222984 NCT05564650 NCT01087151
APG‐1252	BCL‐xL/BCL‐2	BH3 mimetic	—	
AZD‐0466	BCL‐xL/BCL‐2	BH3 mimetic	—	NCT04865419 NCT03595059
DT2216	BCL‐xL/BCL‐2	Proteosome dependent protein degradation	Von Hippel–Lindau	NCT04886622
XZ739	BCL‐xL/BCL‐2	Proteosome dependent protein degradation	Cerebron	—
PZ703b	BCL‐xL/BCL‐2	Proteosome dependent protein degradation	Von Hippel–Lindau	—
XZ338	BCL‐xL	Proteosome dependent protein degradation	Von Hippel–Lindau	—

## 9 Conclusions and Future Perspectives

Overexpression and functional dependency on BCL‐xL are emerging as recurrent features of several myeloid malignancies, particularly those with erythroid/megakaryocytic differentiation and post‐MPN evolution. Experimental and translational data indicate that BCL‐xL contributes not only to leukaemic cell survival and chemoresistance but also to disease progression, making it an attractive therapeutic target across a spectrum of myeloid neoplasms. However, the central role of BCL‐xL in erythropoiesis and platelet homeostasis has so far limited the clinical exploitation of conventional BCL‐xL inhibitors because of on‐target cytopenias, especially thrombocytopenia.

The development of more sophisticated approaches, including modified BH3 mimetics, dendrimer‐based formulations, antibody–drug conjugates and more recently, PROTAC‐based degraders and IAP‐recruiting SNIPERs, is beginning to reshape this scenario. These strategies aim to widen the therapeutic window by achieving preferential targeting of BCL‐xL in malignant cells while sparing platelets and normal haematopoietic progenitors. Early preclinical and clinical data with agents such as navitoclax combinations and the BCL‐xL degrader DT2216 support the feasibility of this concept and suggest that selective elimination of BCL‐xL‐dependent clones may be particularly relevant in high‐risk settings such as JAK2‐mutated post‐MPN AML.

Looking ahead, several questions remain central for the rational clinical development of BCL‐xL‐directed therapies. Robust biomarkers are needed to identify BCL‐xL addiction in individual patients and to guide treatment selection, alone or in combination with BCL‐2 inhibitors, JAK inhibitors, hypomethylating agents, or chemotherapy. A better understanding of mechanisms of primary and secondary resistance to BCL‐xL inhibition or degradation will be essential to optimise dosing, scheduling, and combination partners. In parallel, further refinement of cell‐type–restricted delivery platforms and selective engagement of E3 ligases or IAPs expressed at low levels in platelets may help to further reduce hematologic toxicity.

In conclusion, BCL‐xL has moved from being a biologic hallmark of apoptosis regulation to a concrete therapeutic target in myeloid malignancies. The integration of next‐generation BCL‐xL inhibitors and degraders into clinical practice will require carefully designed trials that balance efficacy with preservation of normal haematopoiesis. If these challenges can be addressed, BCL‐xL‐directed strategies have the potential to expand the armamentarium against myeloid neoplasms and to improve outcomes, particularly in disease subsets that remain difficult to treat with current standards of care.

## Author Contributions

The first draft of the manuscript was written by D.C. and all authors commented on previous versions of the manuscript. All authors read and approved the final manuscript.

## Funding

The authors have nothing to report.

## Conflicts of Interest

The authors declare no conflicts of interest.

## Data Availability

Data sharing not applicable to this article as no datasets were generated or analysed during the current study.
